# Response of Anatomical Structure and Active Component Accumulation in *Apocynum venetum* L. (Apocynaceae) Under Saline Stress and Alkali Stress

**DOI:** 10.3390/plants14142223

**Published:** 2025-07-18

**Authors:** Yanlei Zhang, Shaowei Hu, Xiaxia Wang, Jie Yue, Dongmei Chen, Mingzhi Han, Wanmin Qiao, Yifan Wang, Haixia Wang

**Affiliations:** 1 College of Grassland Science, Qingdao Agricultural University, Qingdao 266109, China; 15092488020@163.com (Y.Z.); 19050518603@163.com (S.H.); wangry_1029@126.com (X.W.); 13176548562@163.com (J.Y.); mzh0101163@163.com (M.H.); qwmzcr@163.com (W.Q.); wyf13066051785@163.com (Y.W.); 2 Shandong Key Laboratory for Germplasm Innovation of Saline-Alkaline Tolerant Grasses and Trees, Qingdao 266109, China; 3 Agriculture and Rural Affairs Bureau of Zhangqiu District, Jinan 250200, China; cdm521@sina.com

**Keywords:** *Apocynum venetum* L., saline stress and alkali stress, biomass, phenotype observation, physiological response

## Abstract

Soil salinization, affecting approximately 954 million hectares globally, severely impairs plant growth and agricultural productivity. *Apocynum venetum* L., a perennial herbaceous plant with ecological and economic value, demonstrates remarkable tolerance to saline and alkali soils. This study investigated the effects of saline (NaCl) and alkali (Na_2_CO_3_ and NaHCO_3_) stress on the growth, anatomical adaptations, and metabolite accumulation of *A. venetum* (*Apocynum venetum* L.). Results showed that alkali stress (100 mM Na_2_CO_3_ and 50 mM NaHCO_3_) inhibited growth more than saline stress (NaCl 240 mM), reducing plant height by 29.36%. Anatomical adaptations included a 40.32% increase in the root cortex-to-diameter ratio (100 mM Na_2_CO_3_ and 50 mM NaHCO_3_), a 101.52% enlargement of xylem vessel diameter (NaCl 240 mM), and a 68.69% thickening of phloem fiber walls in the stem (NaCl 240 mM), enhancing water absorption, salt exclusion, and structural support. Additionally, leaf palisade tissue densification (44.68% increase at NaCl 160 mM), along with epidermal and wax layer adjustments, balanced photosynthesis and water efficiency. Metabolic responses varied with stress conditions. Root soluble sugar content increased 49.28% at NaCl 160 mM. Flavonoid accumulation in roots increased 53.58% at Na_2_CO_3_ 100 mM and NaHCO_3_ 50 mM, enhancing antioxidant defense. However, chlorophyll content and photosynthetic efficiency declined with increasing stress intensity. This study emphasizes the coordinated adaptations of *A. venetum*, providing valuable insights for the development of salt-tolerant crops.

## 1. Introduction

Soil salinization, a significant abiotic stressor, hampers crop growth and productivity [1]. The United Nations Food and Agriculture Organization (FAO) estimates that saline-alkaline land, covering approximately 954 million hectares worldwide (about 7% of the Earth’s land surface), is expanding at a rate of 1 to 1.5 million hectares annually [2,3]. This phenomenon represents and poses a formidable global challenge to sustainable agricultural development [4,5]. Understanding the damage caused by saline stress and alkali stress, as well as plant responses and tolerance mechanisms, is crucial for improving crop resilience and rehabilitating saline and alkali land.

Saline and alkali stress affect the physiological and biochemical processes of plants, thereby causing adverse effects that lead to reduced growth, senescence, and even death [6,7]. In the short term, saline stress reduces water use efficiency, triggering osmotic stress that impairs plant growth [8,9]. Over time, it leads to ion toxicity, causing deficiencies in essential nutrients like Ca^2+^ and K^+^, as well as nutrient imbalances. Additionally, reactive oxygen species (ROS)-mediated oxidative stress exacerbates damage to plant growth [10,11]. The generation of reactive oxygen species (ROS) inhibits a plant’s growth because it interferes with photosynthesis, respiration, starch metabolism, and nitrogen fixation, among other physiological processes [12]. Alkali stress increases the pH on the basis of salt stress. Therefore, in addition to ionic toxicity and osmotic stress, high pH will severely disturb cell pH stability, destroy cell membrane integrity, and decrease root vitality and photosynthetic function [13].

Plants respond to stress through structural adjustments, osmotic regulation, antioxidant responses, hormonal regulation, and the accumulation of secondary metabolites, enhancing adaptability and mitigating adverse effects [14]. For instance, salt stress leads to an increase in the root-to-shoot ratio and enhanced root development, which helps optimize water uptake and ion exclusion [15]. Additionally, plants accumulate compatible solutes to maintain osmotic balance and protect cellular structures from salt-induced damage [16]. Furthermore, plants increase the accumulation of secondary metabolites like flavonoids under salt stress, which have antioxidant properties that help alleviate oxidative stress and improve salt tolerance [17].

*Apocynum venetum* L. (Apocynaceae) is a perennial herb adapted to high-salinity soils (>1.5% salt content), offering ecological, medicinal, and economic benefits [18,19]. It is rich in flavonoids and phenolic acids, and its high-quality stem fibers have earned it the title “King of Wild Fibers” [20]. *A. venetum* (*Apocynum venetum* L.) is widely utilized in high-end textiles, pharmaceuticals, food, and biomaterials, while also serving as a pioneer species in soil and water conservation, polluted farmland remediation, and sustainable saline and alkali land management [21,22]. However, despite its significance, the integrated response of its morphological structures and secondary metabolism to saline stress and alkali stress remains poorly understood.

This study investigates the adaptations of *A. venetum* to saline and alkali stress through a comprehensive analysis of morphological and anatomical changes in roots, stems, and leaves. It also examines the dynamics of metabolites and evaluates organ-specific metabolite accumulation and antioxidant functions. The findings aim to reveal the stress resistance traits of *A. venetum* and provide technical support for its multi-sectoral applications in saline and alkali regions.

## 2. Materials and Methods

### 2.1. Materials

Seeds of *A. venetum* were sourced from Maigaiti County, Kashgar Prefecture, Xinjiang Province. The experiment was conducted in a smart greenhouse at Qingdao Agricultural University. Plants were grown in a 1:1 sand-soil mixture and maintained with 50% Hoagland’s solution weekly. Growth conditions were regulated at 25 ± 3 °C (16 h light)/20 ± 3 °C (8 h dark) with 60–70% relative humidity. After 7 weeks of growth, seedlings with uniform development were selected for the saline and alkali stress treatments. Each treatment group consisted of 90 seedlings, with individual plants cultivated in separate pots to prevent competition and ensure adequate experimental material for all planned analyses.

### 2.2. Methods

#### 2.2.1. Saline Stress and Alkali Stress Treatment

In this study, seven treatment groups were established to investigate plant responses to different stress conditions. The control group was maintained with 50% Hoagland’s nutrient solution throughout the experiment. For saline stress treatments, 80 mM, 160 mM, and 240 mM NaCl were respectively added to 50% Hoagland’s solution. Three alkaline stress treatments were configured in 50% Hoagland’s solution, including a 50 mM Na_2_CO_3_/100 mM NaHCO_3_ mixture (pH 10.0), a 100 mM Na_2_CO_3_/50 mM NaHCO_3_ mixture (pH 10.6), and a 150 mM Na_2_CO_3_ solution (pH 11.7), referred to as Na_2_CO_3_/NaHCO_3_ 50/100 mM, Na_2_CO_3_/NaHCO_3_ 100/50 mM, and Na_2_CO_3_ 150 mM, respectively. To mitigate osmotic shock, seedlings were irrigated daily with a 50% Hoagland’s solution containing NaCl or a Na_2_CO_3_/NaHCO_3_ mixture. The stress treatments were applied continuously for 9 days to reach the expected stress concentrations. Once the target concentrations were reached, the stress treatments were maintained for an additional 14 days, during which the seedlings were irrigated weekly with 50% Hoagland’s solution. Due to complete mortality in plants exposed to 150 mM Na_2_CO_3_, this treatment group was excluded from further analyses. CK is the control group; S1, S2, and S3 are NaCl treatment groups at 80, 160, and 240 mM, respectively; A1 and A2 represent the Na_2_CO_3_/NaHCO_3_ treatment groups at 50/100 and 100/50 mM, respectively.

#### 2.2.2. Growth Parameters Assessment

Growth parameters were assessed using three independent plants randomly selected from each treatment group. Plant height and root development were measured. Fresh weight (FW) of roots, stems, and leaves was determined using an analytical balance. Dry weight (DW) was obtained after two-step drying (105 °C for 10 min, followed by 80 °C until constant mass). Water content was calculated as:

$$\mathrm{Water}\mathrm{content}(\%)=\frac{FW-DW}{FW}\times 100\%$$

#### 2.2.3. Anatomical Features Determination

Root, stem, and leaf samples were collected from the same positions of three seedlings in each treatment group to ensure consistency between treatment groups. Root segments (5 mm) were excised from the middle section of the primary root. Stem segments (5 mm) were collected from the second to third internodes below the shoot apex. Leaf strips (5 mm wide) were excised from the third node below the apex along the midrib. Samples were fixed in FAA (10% formaldehyde, 50% ethanol, 5% acetic acid, *v*/*v*) solution for 48 h, followed by dehydration, clearing, embedding, and sectioning. Paraffin sections (10 µm thick) were stained with safranin-fast green, sealed with neutral resin, and examined under a light microscope. Anatomical features of roots, stems, and leaves were systematically measured. Root measurements included diameter, epidermis thickness, cortex thickness, stele Diameter, xylem and phloem dimensions, and maximum vessel diameter. Stem anatomical parameters comprised diameter, cortex and phloem thicknesses, phloem fiber cell wall thickness, endogenous phloem dimensions, xylem architecture, maximum vessel diameter, and pith diameter. Leaf measurements covered blade thickness, upper and lower epidermal thicknesses, adaxial and abaxial cuticular wax layer thicknesses, spongy and palisade mesophyll thicknesses, xylem and phloem dimensions, and midvein diameter. All measurements were performed in triplicate using ZEN 3.80 software (ZEN Blue Lite, Carl Zeiss AG, Oberkochen, Germany) under three randomly selected microscopic fields of view, with data recorded in micrometers (μm).

#### 2.2.4. Scanning Electron Microscopy (SEM) Observation of Leaf Surface Structures

Leaf samples were collected from the third node below the shoot apex and cut into 5 mm wide segments along the midrib. The segments were fixed in 2.5% glutaraldehyde, rinsed with phosphate buffer, and dehydrated using a graded acetone series. The dehydrated samples underwent critical point drying with carbon dioxide, and were subsequently gold-coated for SEM (JSM-IT500, JEOL Japan Electronics Co., Ltd., Tokyo, Japan) observation [23]. The number of papillae per unit area on the leaf surface was quantified using Image J software (Version 1.53f, National Institutes of Health, Bethesda, MD, USA) by randomly selecting three microscopic fields of view for analysis.

#### 2.2.5. Physiological Parameters Measurement

The contents of soluble proteins, soluble sugars, and total flavonoids were determined in roots, stems, and leaves. Additionally, chlorophyll content, carotenoid content, and photosynthetic efficiency (Fv/Fm) were measured in leaves. All physiological parameters were measured in triplicate across all treatment groups.

1. Soluble Protein Determination;

Protein content was determined using Coomassie Brilliant Blue G-250 staining [24]. Fresh tissue samples (0.5 g) were homogenized in 5 mL of phosphate buffer solution (pH 7.8) and centrifuged at 5000× *g* for 10 min. The supernatant (1 mL) was reacted with 5 mL of Coomassie Brilliant Blue solution (0.1 g·L^−1^). After incubation at ambient temperature for 5 min, spectrophotometric absorbance was measured at 595 nm. Protein levels were quantified using a bovine serum albumin (BSA) calibration curve.

2. Soluble Sugar Determination;

Soluble sugar content was measured using the anthrone colorimetric method [25]. Fresh tissue (0.1 g) was homogenized in 0.5 mL distilled water, extracted twice in boiling water (30 min each), filtered, and adjusted to 25 mL. For analysis, 0.5 mL anthrone-ethyl acetate reagent and 5 mL concentrated sulfuric acid were added, mixed, and heated (1 min, boiling water). After cooling, absorbance at 630 nm was measured, and sugar content was calculated using a sucrose standard curve.

3. Total Flavonoid Determination;

Total flavonoid content was determined using the sodium nitrite-aluminum nitrate colorimetric method [26,27]. Dried tissue samples (0.2 g) were extracted in 20 mL of 70% ethanol via ultrasonic treatment at 50 °C for 40 min, centrifuged (5000× *g*, 10 min), and the supernatant was collected. The extraction was repeated five times, and the final volume was adjusted to 100 mL. For analysis, 0.4 mL of 5% sodium nitrite was added, followed by 0.4 mL of 10% aluminum nitrate, with a 5 min incubation after each addition. Subsequently, 4 mL of 4% sodium hydroxide was introduced, and the solution was diluted to 10 mL with distilled water. After thorough mixing, the reaction proceeded for 15 min, and absorbance at 510 nm was measured. Flavonoid content was quantified using a standard curve.

4. Photosynthetic Efficiency Determination (Fv/Fm);

Photosynthetic efficiency (Fv/Fm) was measured using a PSI portable fluorometer (FluorPenFP110, Photon Systems Instruments, Brno, Czech Republic). Leaves were dark-adapted for 30 min before readings were taken.

5. Chlorophyll and Carotenoid Contents Determination;

Leaf pigments were analyzed following the Lichtenthaler [28]. Leaf tissue (0.3 g) was finely sectioned and extracted in 15 mL of 95% ethanol under dark conditions for 12 h. The extract’s absorbance was measured at 663 nm, 646 nm, and 470 nm using a spectrophotometer (UV-2600i Plus, Shimadzu Corporatio, Kyoto, Japan). The following equations were used to calculate pigment concentrations.

$$\mathrm{Chlorophyll}a\mathrm{content}(\mathrm{Ca},\;\mathrm{mg}\cdot g^{-1})=(13.95\times A_{665}-6.88\times A_{699})\frac{V}{W\times 1000}$$

$$\mathrm{Chlorophyll}b\mathrm{content}(\mathrm{Cb},\;\mathrm{mg}\cdot g^{-1})=(24.96\times A_{649}-7.32\times A_{665})\frac{V}{W\times 1000}$$

Total chlorophyll content = Ca + Cb

$$\mathrm{Carotenoid}\;\mathrm{content}\;(\mathrm{Cc},\;\mathrm{mg}\cdot g^{-1})=(1000\times A_{470}-2.05\times C_{a}-114.8\times C_{b})\frac{V}{W\times 1000}$$

#### 2.2.6. Statistical Analysis

The Tukey multiple comparison test (*p* < 0.05) was used to calculate the statistical significance between the different treatment groups by SPSS 26.0 software (IBM, Armonk, NY, USA). Origin 2021 (OriginLab Corporation, Northampton, MA, USA) and Adobe Photoshop 2019 (Adobe Inc., San Jose, CA, USA) software were used to segregate data and create graphs.

## 3. Results

### 3.1. Effects of Saline and Alkali Stress on the Growth of A. venetum

**Figure 1:**
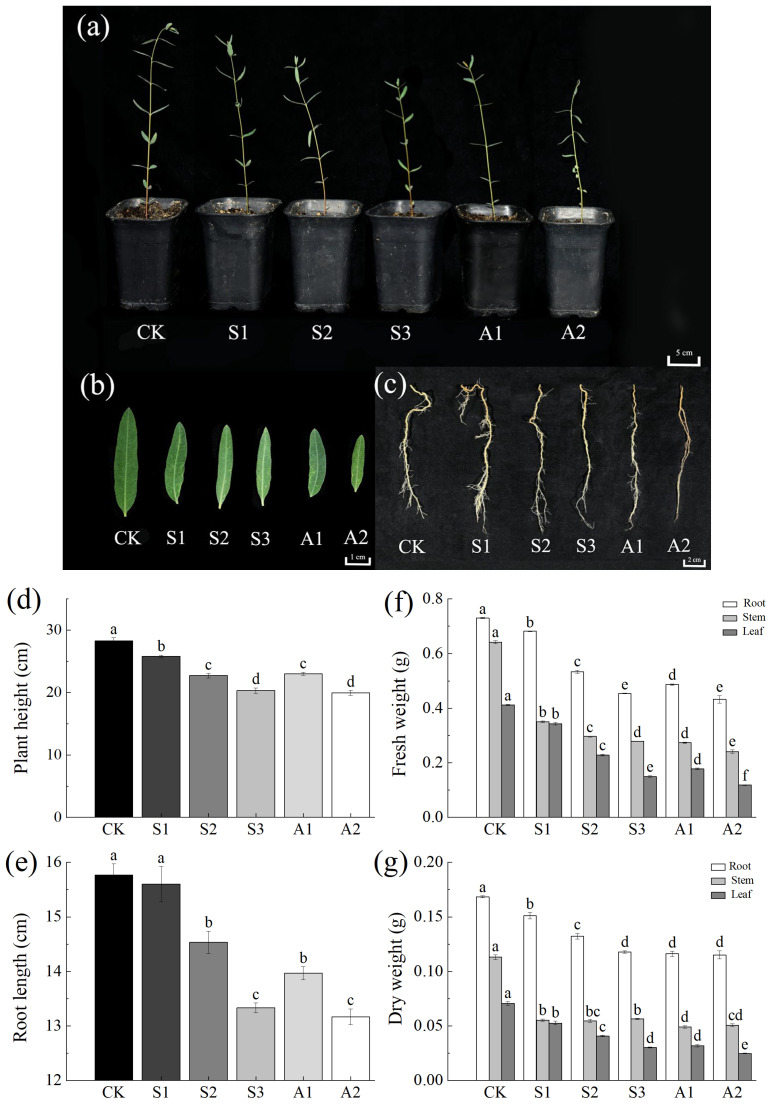
Biomass changes in *A. venetum* (*Apocynum venetum* L.) under saline stress and alkali stress. (**a**) Stem and leaf morphology; (**b**) Leaf appearance; (**c**) Root system characteristics; (**d**) Plant height; (**e**) Root length; (**f**) Fresh weights of roots, stems, and leaves; (**g**) Dry weights of roots, stems, and leaves. Treatments: CK: Control group; S1: NaCl 80 mM; S2: NaCl 160 mM; S3: NaCl 240 mM; A1: Na_2_CO_3_/NaHCO_3_ 50/100 mM; A2: Na_2_CO_3_/NaHCO_3_ 100/50 mM. Data are presented as the mean ± standard error (SE) of three replicates. Significant differences among treatments, based on ANOVA and multiple range tests, are indicated by non-overlapping letters (a–f) at the 95% confidence level.

The growth of *A. venetum* exhibited significant sensitivity to saline stress and alkali stress, with growth parameters negatively correlated with stress intensity. Alkaline conditions imposed more severe inhibitory effects than saline treatments. Control plants achieved the tallest height (28.27 cm), significantly outperforming all stress-exposed groups (Figure 1a,d). Plants exposed to a Na_2_CO_3_/NaHCO_3_ 100/50 mM exhibited pronounced chlorosis and the greatest reduction in growth, with a height of only 19.97 cm.

As saline stress and alkali stress intensified, the detrimental effects on root development became increasingly apparent (Figure 1c,e). At low saline stress levels (NaCl 80 mM), the number of lateral roots increased. However, with higher stress intensity, lateral root numbers declined, the primary root shortened, and leaves became smaller and chlorotic (Figure 1b).

Biomass, evaluated as fresh and dry weights, served as a key indicator of growth performance. Increasing salt concentrations caused a significant reduction in the fresh and dry weights of roots, stems, and leaves (Figure 1f,g). Under NaCl 240 mM, fresh weights decreased by 37.82%, 56.43%, and 63.66%, while dry weights declined by 29.97%, 50.08%, and 57.09%, respectively. Under the Na_2_CO_3_/NaHCO_3_ 100/50 mM, fresh weights dropped by 40.73%, 62.29%, and 71.21%, with dry weights decreasing by 31.62%, 55.08%, and 64.89%, respectively.

**Figure A1:**
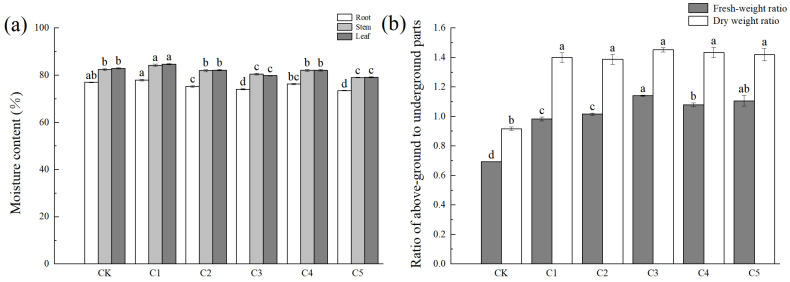
(**a**) Water content of roots, stems, and leaves; (**b**) Ratios of fresh and dry weight between below-ground and above-ground parts. Treatments: CK: Control group; S1: NaCl 80 mM; S2: NaCl 160 mM; S3: NaCl 240 mM; A1: Na_2_CO_3_/NaHCO_3_ 50/100 mM; A2: Na_2_CO_3_/NaHCO_3_ 100/50 mM. Data are presented as the mean ± SE of three replicates. Significant differences among treatments are indicated by non-overlapping letters (a–d).

Interestingly, at NaCl 80 mM, root, stem, and leaf water content increased slightly. However, at NaCl 240 mM and the Na_2_CO_3_/NaHCO_3_ 100/50 mM, water content significantly declined (Figure A1a). The fresh-to-dry weight ratio of below-ground tissues increased under saline stress and alkali stress, indicating preferential organic matter accumulation in roots (Figure A1b).

### 3.2. Measurement and Analysis of Anatomical Features Under Saline and Alkali Stress

#### 3.2.1. Anatomical Variations in the Roots

**Figure 2:**
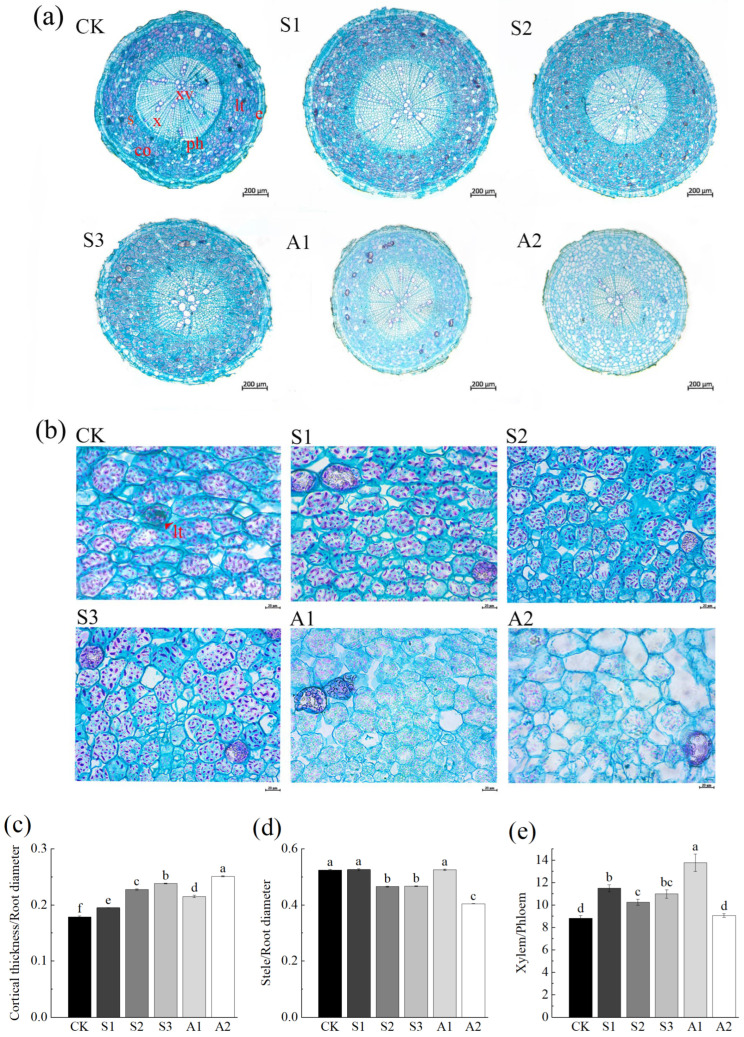
Anatomical structure and parameter ratios of *A. venetum* roots in transverse sections under saline and alkali stress. (**a**) Root cross-section under saline stress and alkali stress (magnification ×40, scale bar = 200 μm); (**b**) Cortex structure (magnification ×400, scale bar = 20 μm). Abbreviations: e: Epidermis; co: Cortex; lt: Laticiferous tube; s: Starch Granule; ph: Phloem; x: Xylem; xv: Xylem Vessel; (**c**) Cortex/Root Diameter: ratio of cortex thickness to root diameter; (**d**) Stele/Root Diameter: ratio of stele diameter to root diameter; (**e**) Xylem/Phloem: ratio of xylem thickness to phloem thickness. Treatments: CK: Control group; S1: NaCl 80 mM; S2: NaCl 160 mM; S3: NaCl 240 mM; A1: Na_2_CO_3_/NaHCO_3_ 50/100 mM; A2: Na_2_CO_3_/NaHCO_3_ 100/50 mM. Data are presented as the mean ± standard error (SE) of three replicates. Significant differences among treatments, based on ANOVA and multiple range tests, are indicated by non-overlapping letters (a–f) at the 95% confidence level.

In the transverse section of *A. venetum* roots, three distinct regions are evident: the epidermis, cortex, and stele, organized from the exterior to the interior. Laticiferous tubes are scattered throughout the cortex. The stele occupies a significant portion of the root, characterized by well-developed xylem with large vessel diameters, while the phloem occupies a comparatively smaller proportion (Figure 2a).

The anatomical parameters of *A. venetum* roots under saline stress and alkali stress were analyzed (Table A1). Under NaCl 0–240 mM, epidermis thickness initially increased and then decreased, while in the Na_2_CO_3_/NaHCO_3_ 50/100 mM and Na_2_CO_3_/NaHCO_3_ 100/50 mM treatment groups, epidermis thickness significantly decreased compared to the control group. Cortex thickness increased in response to stress, with the cortex-to-root diameter ratio rising by 33.14% at NaCl 240 mM and 40.32% at Na_2_CO_3_/NaHCO_3_ 100/50 mM. Conversely, the stele-to-root diameter ratio decreased, dropping by 11.25% at NaCl 160 mM and 22.96% at Na_2_CO_3_/NaHCO_3_ 100/50 mM. The xylo-phloem ratio increased significantly, rising by 30.35% at NaCl 80 mM and 55.94% at Na_2_CO_3_/NaHCO_3_ 50/100 mM (Table A1).

Additionally, the maximum xylem vessel diameter increased significantly, by 101.52% at NaCl 240 mM and 36.21% at Na_2_CO_3_/NaHCO_3_ 100/50 mM. Paraffin section staining with safranin-fast green revealed variations in the staining intensity of the cortex, and differences in cell density per unit area across saline stress and alkali stress treatments. Under NaCl stress (0–240 mM), cell packing density in the cortex initially increased and then decreased, though it remained higher than the control group even at 240 mM. Similarly, under Na_2_CO_3_/NaHCO_3_ 50/100 mM, the cell packing density was greater than that of the control group (Figure 2). The variation in staining intensity among cortical cells in Figure 2a is likely associated with the contents of carbohydrates and proteins within these cells.

#### 3.2.2. Anatomical Variations in the Stem

The stem of *A. venetum* comprises five primary regions: the epidermis, cortex, phloem, xylem, and pith. The cortical parenchyma cells are notably large, densely packed, and exhibit minimal intercellular spaces. Scattered throughout the cortex are laticifers. Beneath the cortex lies the vascular cylinder, with the outer region containing abundant phloem fibers.

At the center of the stem, the pith is composed of large parenchyma cells interspersed with numerous laticifers. Surrounding the periphery of the pith, adjacent to the protoxylem, is an inner ring of intraxylary phloem. This unique anatomical structure, including the presence of laticifers and densely packed cortical cells, contributes to the stem’s structural stability and functionality under saline stress and alkali stress.

Under saline stress and alkali stress, the cortex-to-stem diameter ratio in *A. venetum* increased significantly. This ratio rose by 20.76% under NaCl 240 mM and 51.88% under Na_2_CO_3_/NaHCO_3_ 50/100 mM compared to the control. The proportion of xylem also increased, with an increment of 16.10% under NaCl 160 mM and 21.28% under Na_2_CO_3_/NaHCO_3_ 100/50 mM. Furthermore, the maximum xylem vessel diameter expanded by 54.18% under NaCl 240 mM and 32.53% under Na_2_CO_3_/NaHCO_3_ 100/50 mM. These changes may enhance water and nutrient transport efficiency while improving the structural integrity of the stem (Table A2).

The pith, which plays a crucial role in support, vascular conduction, and storage, exhibited a significant increase in the pith-to-stem diameter ratio under saline stress and alkali stress. When extraxylary phloem was damaged, the intraxylary phloem compensated, facilitating adaptation to drought and saline and alkali conditions. Under NaCl 0–240 mM, the intraxylary phloem remained stable, while under mixed Na_2_CO_3_ and NaHCO_3_ stress (50/50 mM and 100/50 mM), its proportion increased significantly. Additionally, the xylem-to-phloem ratio showed a notable increase under stress conditions compared to the control.

**Figure 3:**
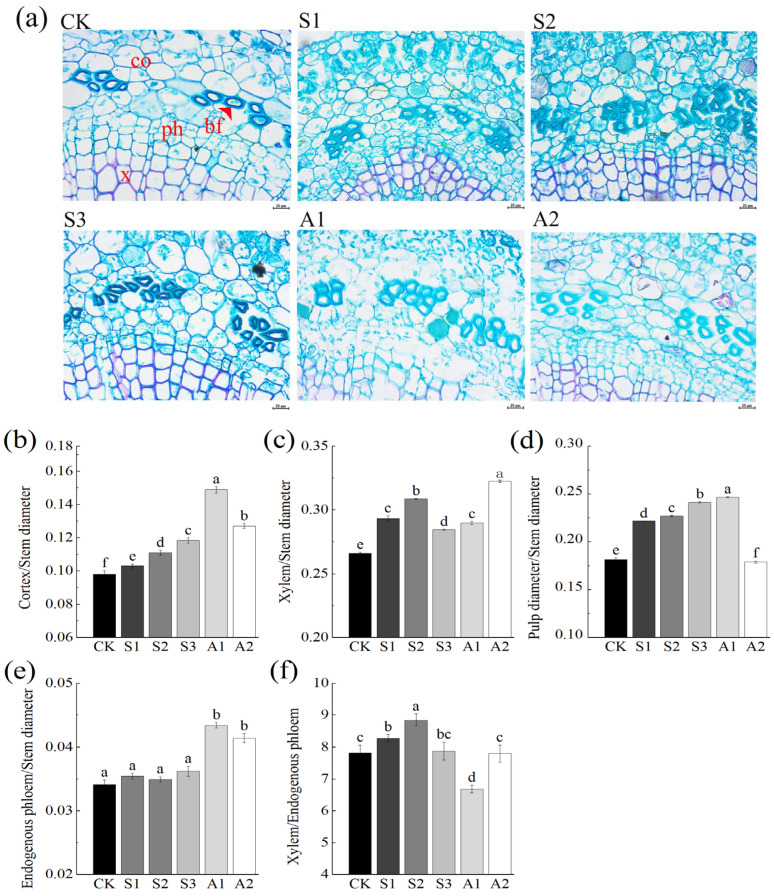
Anatomical structure and parameter ratios of *A. venetum* stems in transverse sections under saline and alkali stress. (**a**) Transverse stem structure of *A. venetum* under different saline stress and alkali stress treatments (magnification ×400, scale bar = 20 μm). Abbreviations: bf: Phloem fiber cells; co: Cortex; x: Xylem; Ph: Phloem; (**b**) Cortex/Stem Diameter: ratio of cortex thickness to stem diameter; (**c**) Xylem/Stem Diameter: ratio of xylem thickness to stem diameter; (**d**) Pulp Diameter/Stem Diameter: ratio of pith diameter to stem diameter; (**e**) Endogenous Phloem/Stem Diameter: ratio of intraxylary phloem thickness to stem diameter; (**f**) Xylem/Endogenous Phloem: ratio of xylem thickness to Endogenous Phloem. Treatments: CK: Control Group; S1: NaCl 80 mM; S2: NaCl 160 mM; S3: NaCl 240 mM; A1: Na_2_CO_3_/NaHCO_3_ 50/100 mM; A2: Na_2_CO_3_/NaHCO_3_ 100/50 mM. The presented values represent the mean ± standard error (SE) based on three replicates. Non-overlapping letters (a–f) indicate significant differences among treatments, determined through ANOVA and multiple range tests at a 95% confidence level.

The phloem fibers of *A. venetum* are located within the cortex and between vascular bundles. These fibers are composed of elongated, spindle-shaped cells with tapered ends, thickened cell walls, and narrow, elongated lumens. Saline stress significantly increased the thickness of the phloem fiber cell wall compared to the control group. Under NaCl 240 mM, cell wall thickness increased by 68.69%. However, under treatment with Na_2_CO_3_/NaHCO_3_ 100/50 mM, the cell wall thickness decreased by 61.98%, which may be associated with structural damage in the plant (Figure 3a). These phloem fibers play a vital role in enhancing the plant’s mechanical strength, providing resistance to breakage and bending.

#### 3.2.3. Anatomical Variations in the Leaf

**Figure 4:**
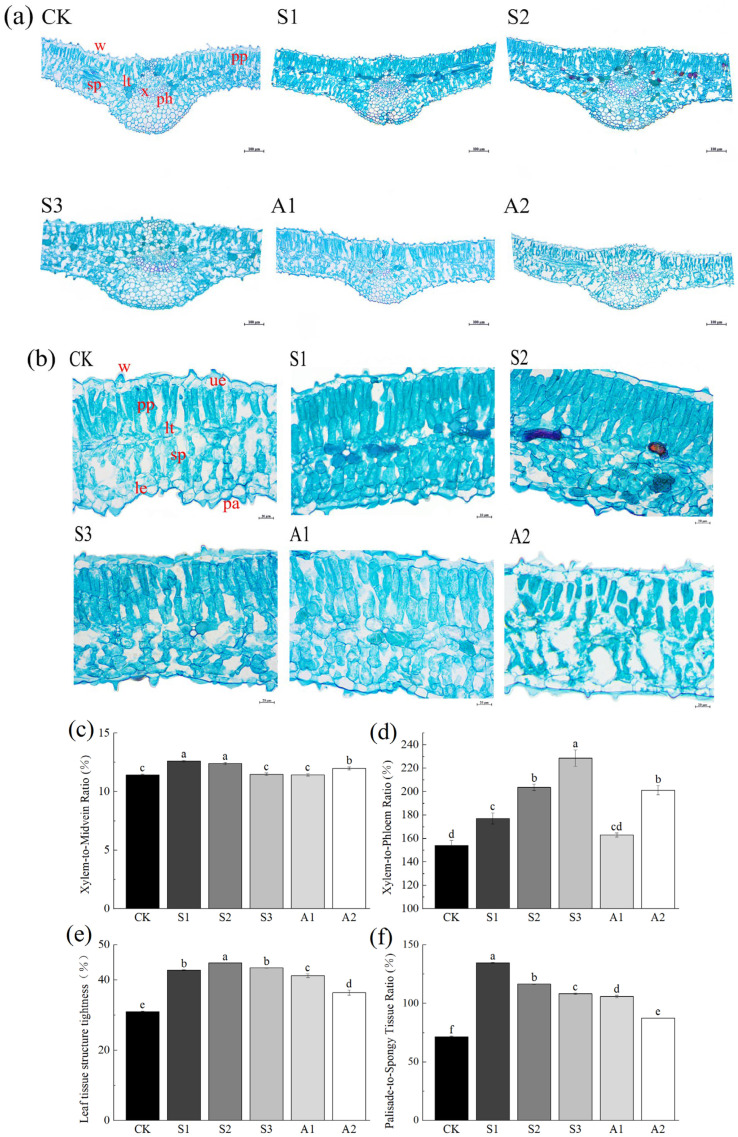
Anatomical structure and parameter ratios of *A. venetum* leaves in transverse sections under saline and alkali stress. (**a**) Cross-sectional view of *A. venetum* leaves under different saline stress and alkali stress treatments (magnification ×100, scale bar = 100 μm); (**b**) Transverse mesophyll structure of *A. venetum* leaves under different saline stress and alkali stress treatments (magnification ×400, scale bar = 20 μm). Abbreviations: ue: Upper epidermis; le: Lower epidermis; pp: Palisade tissue; sp: Spongy tissue; x: Xylem; ph: Phloem; lt: Laticifer; pa: Papillae; w: Wax layer. (**c**) Xylem-to-Midvein Ratio = (Xylem Thickness/Midvein Diameter) × 100%; (**d**) Xylem-to-Phloem Ratio = (Xylem Thickness/Phloem Thickness) × 100%; (**e**) Leaf tissue structure tightness = (Palisade Tissue Thickness/Leaf Thickness) × 100%; (**f**) Palisade-to-Spongy Tissue Ratio = (Palisade Tissue Thickness/Spongy Tissue Thickness) × 100%. Treatments: CK: Control Group; S1: NaCl 80 mM; S2: NaCl 160 mM; S3: NaCl 240 mM; A1: Na_2_CO_3_/NaHCO_3_ 50/100 mM; A2: Na_2_CO_3_/NaHCO_3_ 100/50 mM. Presented values are the mean ± SE of three replicates. Non-overlapping letters (a–f) indicate significant differences among treatments, determined by ANOVA and multiple range tests at a 95% confidence level.

The leaves of *A. venetum* are relatively thin, with papillae distributed on both the upper and lower epidermal surfaces, covered by a waxy layer. The palisade tissue consists of two layers of densely packed cells, while the spongy tissue is moderately compact. Laticifers are interspersed between the palisade and spongy tissues. The midvein, convex on the lower surface, contains well-developed xylem with numerous large vessels, which facilitate water transport and provide mechanical support (Figure 4a,b).

Under saline stress and alkali stress, the xylem proportion in the midvein increased. In NaCl 80 mM treatments, the xylem-to-midvein ratio was 10.42% higher than in the control. Similarly, the xylem-to-phloem ratio increased by 48.40% under NaCl 240 mM and 30.64% under Na_2_CO_3_/NaHCO_3_ 100/50 mM, reflecting structural adaptations to improve vascular function under stress conditions.

The average leaf thickness of *A. venetum* increased significantly under saline stress (NaCl 0–240 mM). A similar trend was observed at Na_2_CO_3_/NaHCO_3_ 50/100 mM, but leaf thickness decreased by 10.91% at Na_2_CO_3_/NaHCO_3_ 100/50 mM compared to the control. Saline stress and alkali stress promoted mesophyll thickening, mainly due to longitudinal expansion of the palisade tissue. The palisade-to-spongy tissue ratio increased significantly, with an 88.15% rise under NaCl 80 mM. Stress conditions enhanced tissue compactness, reducing intercellular spaces (Table A3).

Under saline and alkali stress, the leaves of *A. venetum* exhibited thinning of the upper epidermis and thickening of the lower epidermis. Specifically, compared to the control conditions, the thickness of the upper epidermis decreased by up to 37.05% in the Na_2_CO_3_/NaHCO_3_ 100/50 mM treatment group, while the thickness of the lower epidermis increased by up to 25.09% in the NaCl 160 mM treatment group. Cuticle thickness varied under stress: it thinned on the upper epidermal papillae but thickened on the lower epidermis. The epidermal wax layer is crucial for radiation resistance, light reflection, and water retention. Thinning of the upper wax layer increased light penetration, improving photosynthetic efficiency, while thickening of the lower wax layer reduced non-stomatal water loss, limiting transpiration and maintaining hydration. In addition, a comparison of leaf structures across different treatment groups revealed that under NaCl 240 mM and Na_2_CO_3_/NaHCO_3_ 100/50 mM stress conditions, the plant leaf tissues showed noticeable structural damage.

The leaves of *A. venetum* feature papillated surfaces that increase the leaf area and provide partial shading for stomata, reducing direct exposure to intense light. Stomata are located on both epidermal layers, with a higher density on the lower epidermis, aiding in the regulation of transpiration and facilitating water vapor absorption.

**Figure 5:**
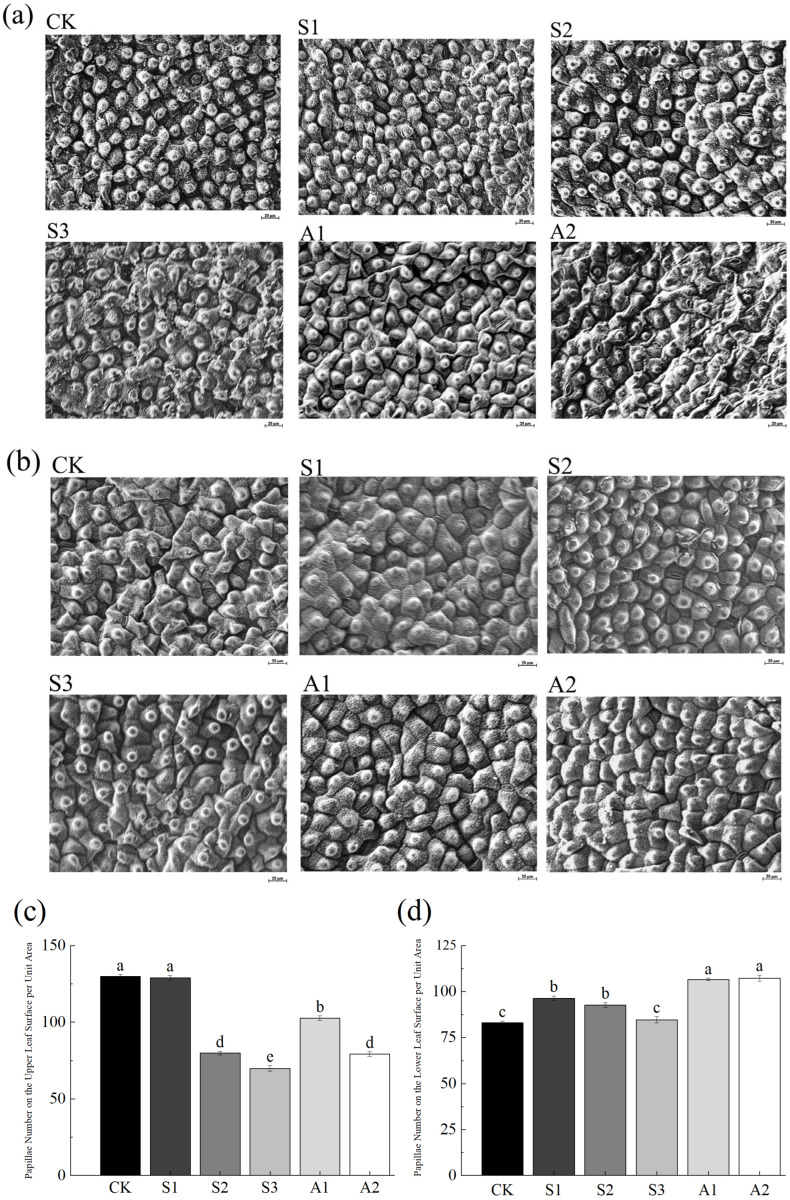
Comparison of Scanning Electron Microscopy (SEM) images of the upper and lower leaf surfaces of *A. venetum* under saline stress and alkali stress. (**a**) Upper Leaf Surface (magnification ×400, scale bar = 20 μm); (**b**) Lower Leaf Surface (magnification ×400, scale bar = 20 μm); (**c**) Papillae Number on the Upper Leaf Surface per Unit Area; (**d**) Papillae Number on the Lower Leaf Surface per Unit Area. Treatments: CK: Control group; S1: NaCl 80 mM; S2: NaCl 160 mM; S3: NaCl 240 mM; A1: Na_2_CO_3_/NaHCO_3_ 50/100 mM; A2: Na_2_CO_3_/NaHCO_3_ 100/50 mM. Presented values are the mean ± SE of three replicates. Non-overlapping letters (a–e) indicate significant differences among treatments, determined by ANOVA and multiple range tests at a 95% confidence level.

Under saline stress and alkali stress (Figure 5), papillae density on the upper epidermis decreased, while it increased on the lower epidermis. This adaptation potentially enhances photosynthetic efficiency by improving light penetration while reducing water loss through transpiration. However, under severe stress, leaf dehydration was observed, characterized by declining water content, tissue deterioration, and shrinkage of the upper epidermis. These changes suggest that *A. venetum* employs structural adaptations to maintain hydration and limit water loss under adverse conditions.

### 3.3. Measurement and Analysis of Photosynthetic Pigment Content and Fluorescence Parameters Under Saline and Alkali Stress

**Figure 6:**
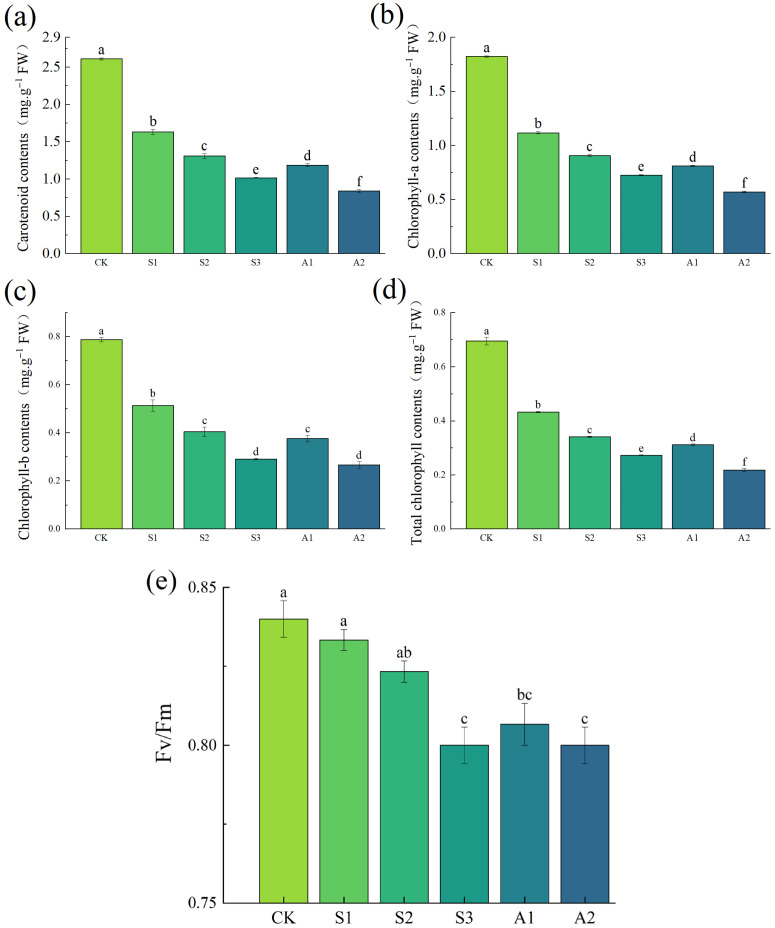
Changes in chlorophyll content and Fv/Fm parameters of *A. venetum* under different saline and alkali stress conditions. (**a**) Total chlorophyll content; (**b**) Chlorophyll a content; (**c**) Chlorophyll b content; (**d**) Carotenoid content; (**e**) Fv/Fm Values. Treatments: CK: Control group; S1: NaCl 80 mM; S2: NaCl 160 mM; S3: NaCl 240 mM; A1: Na_2_CO_3_/NaHCO_3_ 50/100 mM; A2: Na_2_CO_3_/NaHCO_3_ 100/50 mM. Values represent the mean ± SE of three replicates. Non-overlapping letters (a–f) signify significant differences among treatments, based on ANOVA and multiple range tests at a 95% confidence level.

As shown in Figure 6, the chlorophyll content in the leaves of *A. venetum* progressively declined with increasing saline and alkali stress. Both chlorophyll a and b, as well as carotenoid content, exhibited significant reductions as stress intensity increased. Notably, treatments involving NaCl 240 mM and Na_2_CO_3_/NaHCO_3_ 100/50 mM caused a more pronounced decrease in chlorophyll a and carotenoids compared to the control group. Alkaline stress exerted a stronger inhibitory effect on chlorophyll content, particularly on chlorophyll a and carotenoids. These findings suggest that high saline stress and alkali stress suppress chlorophyll synthesis while accelerating its degradation, leading to leaf yellowing and a reduced photosynthetic rate (Figure 1b).

Salt and alkali stress disrupt chloroplast structure, impairing photosynthesis. Under saline stress and alkali stress, the Fv/Fm values—a key indicator of photosynthetic efficiency—were consistently lower than those of the control group and exhibited a progressive decline with increasing stress intensity. Specifically, the Fv/Fm values in the NaCl 240 mM and Na_2_CO_3_/NaHCO_3_ 100/50 mM treatment groups were significantly reduced compared to the control (Figure 6e). This reduction indicates severe damage to the photosynthetic apparatus under high-stress conditions.

### 3.4. Measurement and Analysis of Osmotic Regulation Substances Under Saline and Alkali Stress

**Figure 7:**
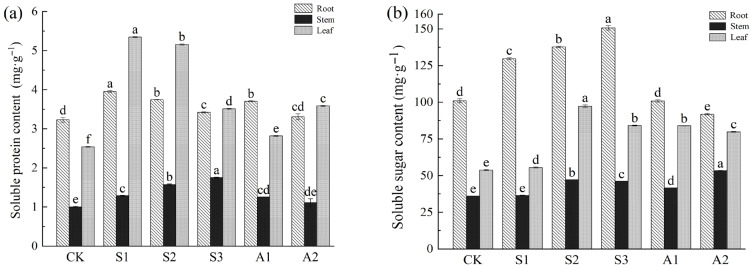
Variations in soluble protein and soluble sugar in the roots, stems, and leaves of *A. venetum* under saline stress and alkali stress. (**a**) Soluble protein content in roots, stems, and leaves; (**b**) Soluble sugar content in roots, stems, and leaves. Treatments: CK: Control group; S1: NaCl 80 mM; S2: NaCl 160 mM; S3: NaCl 240 mM; A1: Na_2_CO_3_/NaHCO_3_ 50/100 mM; A2: Na_2_CO_3_/NaHCO_3_ 100/50 mM. Values are presented as mean ± SE of three replicates. Non-overlapping letters (a–f) indicate significant differences among treatments, based on ANOVA and multiple range tests at a 95% confidence level.

The soluble protein content in the roots initially increased and then decreased with increasing concentrations of NaCl (0–240 mM) and Na_2_CO_3_/NaHCO_3_ mixtures (0, 50/100, 100/50 mM). The highest protein content in roots was observed at 80 mM NaCl (22.44% increase) and Na_2_CO_3_/NaHCO_3_ 50/100 mM (14.66% increase). In stems, protein content showed a continuous increase, peaking at a 75.23% rise under NaCl 240 mM. In leaves, protein content demonstrated variable responses, with the highest increase recorded ate110.58% under NaCl 80 mM (Figure 7a).

Soluble sugar content in roots increased under NaCl stress, with a peak of 49.28% at 160 mM NaCl. However, alkali stress led to a reduction, with an 8.99% decrease observed at Na_2_CO_3_/NaHCO_3_ 100/50 mM. In stems, sugar levels increased by 30.81% at NaCl 160 mM and 47.66% at Na_2_CO_3_/NaHCO_3_ 50/100 mM. Leaves exhibited an initial increase, with the highest sugar content recorded at 80.85% under NaCl 160 mM and 56.10% under Na_2_CO_3_/NaHCO_3_ 50/100 mM. Across all treatments, roots contained the highest sugar content, while stems exhibited the lowest levels of protein and sugar (Figure 7b).

### 3.5. Measurement and Analysis of Total Flavonoid Content Under Saline and Alkali Stress

**Figure 8:**
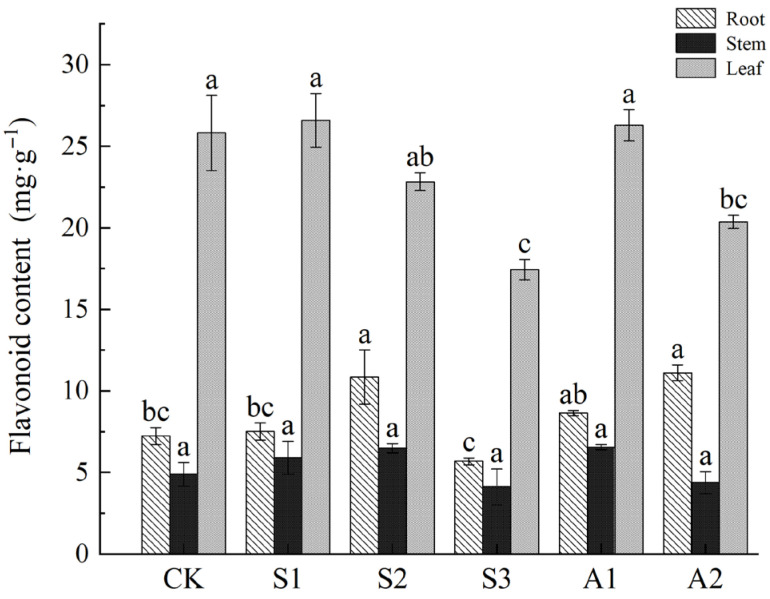
Variations in total flavonoid content in the roots, stems, and leaves of *A. venetum* under saline stress and alkali stress. Treatments: CK: Control group; S1: NaCl 80 mM; S2: NaCl 160 mM; S3: NaCl 240 mM; A1: Na_2_CO_3_/NaHCO_3_ 50/100 mM; A2: Na_2_CO_3_/NaHCO_3_ 100/50 mM. Values are presented as mean ± SE of three replicates. Non-overlapping letters (a–c) indicate significant differences among treatments, based on ANOVA and multiple range tests at a 95% confidence level.

Flavonoids, essential for stress adaptation, were most abundant in leaves, followed by roots and stems. Root flavonoid content increased significantly under stress, peaking at 49.97% under NaCl 160 mM and 53.58% under Na_2_CO_3_/NaHCO_3_ 100/50 mM. In contrast, stem flavonoid levels remained relatively stable. Leaf flavonoid content, however, declined under stress, decreasing by 32.50% under NaCl 240 mM and 21.16% under Na_2_CO_3_/NaHCO_3_ 100/50 mM. The accumulation of flavonoids in roots likely plays a key role in mitigating oxidative stress, thereby contributing to salt tolerance (Figure 8).

## 4. Discussion

### 4.1. Effects of Saline Stress and Alkali Stress on the Growth of A. venetum

This study demonstrates that saline stress and alkali stress significantly influences the growth and development of *A. venetum*. Under saline stress and alkali stress, plants allocate a substantial portion of photosynthetic products to synthesize organic solutes, which are essential for osmotic regulation. However, this allocation reduces the energy available for growth, thereby slowing the plant’s overall growth rate [29,30].

As the severity of saline and alkali stress increased, the fresh and dry weights of *A. venetum* roots, stems, and leaves decreased markedly. Alkaline stress exerted a more pronounced inhibitory effect on growth than salt stress, particularly in above-ground plant parts.

Under saline stress and alkali stress, *A. venetum* adapts by reallocating biomass to prioritize root growth over shoot development, resulting in an increased root-to-shoot ratio. This strategy optimizes resource distribution to below-ground parts, enabling the plant to better cope with high-salinity environments [31]. Similar adaptive mechanisms have been observed in other salt-tolerant species, such as *Chenopodium quinoa* [32] and *Suaeda salsa* [33].

As the primary organ exposed to soil salinity, roots must adapt to external conditions while maintaining efficient nutrient and water uptake to support plant growth [34]. Salt stress typically reduces root biomass and alters the distribution of root structural components, affecting both primary and lateral root growth rates, and inhibiting lateral root formation [35,36].

### 4.2. Adaptive Changes in Anatomical Structures

Adaptation to salt stress in plants is often accompanied by specific morphological and anatomical changes. Root anatomical traits, such as length and diameter, are critical for water and nutrient acquisition to compensate for water loss [37]. As the primary interface between plants and soil, a well-developed root system supports stem growth and enhances overall plant yield [38,39]. Other root traits, including xylem vessel number and diameter, root cortex width, root hair density, and lignin deposition in the root epidermis, influence root water permeability [40]. Under salt stress, an increased root-to-shoot ratio plays a pivotal role in limiting the transport of toxic ions to aerial parts—a typical survival mechanism in many salt-tolerant plants [41,42]. In addition to root adaptations, changes in leaf anatomical traits also influence plant responses to salt stress [43]. Traits such as leaf thickness, intercellular space, cell surface area-to-volume ratio, and tissue density impact the photosynthetic rate (PN). Some plants minimize water loss by reducing transpiration and stomatal conductance, thereby improving leaf water balance, enhancing photosynthetic efficiency, and increasing water use efficiency [44,45]. Under saline stress and alkali stress, *A. venetum* exhibits coordinated structural and functional adaptations in its roots, stems, and leaves, forming an integrated mechanism to mitigate the adverse effects of stress.

#### 4.2.1. Anatomical and Functional Adaptations of Roots

With increasing saline stress and alkali stress, the thickness of the epidermis decreased while cortex thickness increased significantly. This highlights the critical role of the cortex in salt tolerance and adaptation. Cortical cells became densely packed and accumulated osmotic regulatory substances, such as soluble sugars and proteins, which mitigated water loss caused by salt stress through osmotic adjustment. These adaptations were essential for maintaining normal root function.

Additionally, the increased xylem-to-phloem ratio and the enlargement of vessel diameters significantly enhanced the roots’ water transport capacity. Concurrently, the reduction in the stele-to-root diameter ratio limited excessive accumulation of harmful salts [46,47]. This “salt exclusion” mechanism confines salts to the root zone, minimizing translocation to above-ground tissues, a key adaptive strategy in salt-tolerant plants [13,48]. Similar structural adaptations have been reported in other species, such as hybrid walnut (*Juglans major* × *Juglans regia*) [49] and cowpea (*Vigna unguiculata* L.) [50]. The ability to regulate anatomical structures to confine salts to the root zone is critical for maintaining functional integrity under stress conditions.

#### 4.2.2. Enhanced Transport and Support Functions in Stems

As a critical conduit for water and nutrient transport, the stem undergoes significant adaptive modifications under saline stress and alkali stress. In this study, cortical thickness increased substantially, along with significant increases in xylem thickness and the xylem-to-stem diameter ratio.

The thickness of phloem fiber cell walls also increased significantly, enhancing the stem’s ability to withstand stress while maintaining its morphology and structural integrity [51,52]. Additionally, the diameter and proportion of pith tissue expanded notably, with an intraxylary phloem ring forming around its periphery. This specialized structure provides mechanical support and serves as a site for water and nutrient storage and conduction [53]. Similar adaptations have been observed in other species, including *Fraxinus* spp. [54], *Populus euphratica* [55], and *Glycine max* [56].

#### 4.2.3. Adaptation and Optimization of Leaf for Enhanced Photosynthesis

As the primary site of photosynthesis in plants, leaves undergo significant structural adaptations under saline stress and alkali stress to maintain both photosynthetic function and water balance. In this study, while leaf thickness decreased under the Na_2_CO_3_/NaHCO_3_ 100/50 mM treatment, a substantial increase was observed in all other treatment groups. These changes were characterized by a reduction in the thickness of the upper epidermis and an increase in that of the lower epidermis. The palisade tissue exhibited marked thickening and became more densely packed, resulting in a significant increase in the palisade-to-spongy tissue thickness ratio. This compact structure effectively minimized water loss through transpiration while enhancing photosynthetic efficiency, thereby demonstrating the adaptive responses of leaves to saline and alkali conditions [56]. Additionally, the reduced proportion of spongy tissue facilitated more efficient CO_2_ diffusion within the intercellular spaces [57,58]. Such structural modifications have also been reported in the leaves of other salt-tolerant species, including *Salicornia* spp. [59] and *Tamarix* spp. [60].

Our findings reveal that the wax layer on the upper surface of *A. venetum* leaves became thinner, while the cuticle on the lower surface thickened and compacted. The reduction in the thickness of the wax layer decreased light reflection, thereby enhancing light penetration through the epidermis and subsequently boosting photosynthetic activity [61,62]. In contrast, the thickened and compacted cuticle on the lower surface effectively minimized water loss via transpiration, thus improving the leaves’ water retention capacity [63]. This bidirectional regulatory mechanism simultaneously maintained water balance under high salt stress and improved photosynthetic efficiency to a certain extent.

### 4.3. Effects of Saline Stress and Alkali Stress on Photosynthetic Pigments and Photosynthesis

Saline stress and alkali stress significantly reduced the photosynthetic pigment content and photosynthetic efficiency in the leaves of *A. venetum*, with Na_2_CO_3_/NaHCO_3_ causing more severe effects than NaCl. Chlorophyll a, chlorophyll b, and carotenoid contents decreased markedly as saline and alkali concentrations increased, with maximum reductions of 68.70%, 66.14%, and 67.93%, respectively. These reductions in pigment levels led to a significant decline in the photosynthetic efficiency parameter Fv/Fm. Similar trends have been observed in other plant species, such as rice (*Oryza sativa* L.) [64], wheat (*Triticum aestivum* L.) [65], *Arabidopsis thaliana* [66], and cotton (*Gossypium hirsutum* × *Gossypium arboreum* × *Gossypium raimondii*) [12], corroborating the findings of this study.

Saline and alkali stress significantly reduce chlorophyll content, resulting in leaf yellowing and a decline in photosynthetic capacity. The decrease in carotenoid content further destabilizes photosystem II (PSII). These effects are primarily attributed to osmotic stress and ion toxicity induced by high salinity, which collectively impair the plant’s photosynthetic efficiency [67]. Alkaline stress exerts a more pronounced inhibitory effect on photosynthesis. Under alkali stress, the reduction in chlorophyll content and photosynthetic efficiency is more severe, likely due to the negative impact of the alkaline environment on membrane integrity, leading to a decrease in chloroplast content and disruption of cellular pH balance.

Although photosynthesis is inhibited under saline stress and alkali stress, *A. venetum* mitigates these effects through structural adaptations, such as thickening of the palisade tissue and increased mesophyll tissue density. These modifications help maintain a minimum energy supply, ensuring that essential physiological processes can continue to function effectively despite the adverse conditions.

### 4.4. Effects of Saline and Alkali Stress on the Accumulation of Metabolites in A. venetum

To cope with osmotic stress caused by high salinity, plants synthesize osmotic regulatory substances such as soluble sugars, soluble proteins, and betaine. These substances help maintain osmotic balance at the cellular level, preserve cell turgor, and support normal metabolic activities [68]. As osmotic stress intensifies and inhibits water absorption, plants mitigate this pressure by accumulating small molecules of soluble sugars [69]. Plant secondary metabolites also play an essential role in responding to salt stress and alkali stress [70,71]. Flavonoids are key players in combating abiotic stress. For instance, flavonols regulate root development by influencing growth through their antioxidant activity or by modulating auxin transport [72]. Anthocyanins, another group of common flavonoid pigments in plants, play a critical role in oxidative stress resistance. They capture and absorb excess light energy, preventing photodamage to plant leaves under abiotic stress. Additionally, anthocyanins protect antioxidant enzymes, scavenge free radicals, and interact with molecules involved in other signaling pathways, indirectly contributing to the elimination of ROS. Together, these functions significantly enhance the plant’s capacity to withstand oxidative stress [73].

#### 4.4.1. Osmotic Regulation Roles of Soluble Sugars and Soluble Proteins

The contents of soluble sugars and proteins in the roots, stems, and leaves of *A. venetum* exhibit significant changes under saline stress and alkali stress, highlighting the critical role of osmotic regulation in maintaining water balance and mitigating ionic toxicity.

Saline stress and alkali stress induced dynamic changes in soluble sugar levels. The roots exhibited significantly higher soluble sugar content than the stems and leaves, indicating that the roots serve as the primary defense mechanism against saline stress and alkali stress. Under NaCl stress, root sugar levels initially increased but declined with increasing concentrations. Conversely, under alkali stress, sugar content continuously decreased, suggesting that high-concentration alkali stress inhibits root glucose metabolism. This decline may be closely linked to damage to root cell membrane integrity.

In contrast, the accumulation of soluble sugars in leaves and stems increased significantly under saline stress and alkali stress. Notably, under the NaCl 160 mM treatment, leaf sugar content increased by 80.85% compared to the control. This accumulation enhances osmotic potential, maintains cell turgor, and alleviates structural damage caused by stress [74].

There is a distinct accumulation pattern of soluble proteins in *A. venetum* under saline stress and alkali stress, emphasizing their critical role in regulating cellular osmotic potential and enhancing antioxidant defenses. The soluble protein content in both roots and stems increased significantly under saline stress and alkali stress. This increase highlights their importance in maintaining osmotic balance and supporting the plant’s stress tolerance mechanisms. Under the NaCl 240 mM treatment, the soluble protein content in stems increased by 75.23% compared to the control, underscoring the enhanced role of stems in material transport and storage under salt stress.

In leaves, soluble protein content exhibited a dynamic trend, initially increasing under moderate stress and then decreasing as stress levels escalated. Specifically, under the NaCl 80 mM treatment, soluble protein content in leaves increased by 110.58%. However, at higher concentrations (240 mM), protein content declined markedly, likely due to accelerated protein degradation caused by excessive salt levels.

#### 4.4.2. Antioxidant Role of Flavonoid Compounds

Flavonoid compounds, as key secondary metabolites, exhibit significant antioxidant functions in *A. venetum* under saline and alkali conditions [72,75].

Flavonoid enrichment in the roots is particularly notable, with a 49.97% increase observed under NaCl 160 mM treatment. This indicates that the roots serve as the primary site for antioxidant defense. The accumulation of flavonoids in roots aids in scavenging reactive oxygen species (ROS) produced under salt stress and mitigating cellular oxidative damage. This adaptation enhances the plant’s salt tolerance, highlighting the critical role of roots in stress mitigation.

Flavonoid content in stems remained relatively stable, reflecting the low secondary metabolic activity in this organ. Stems primarily function in material transport and storage, rather than active metabolic responses to stress.

The accumulation of flavonoid compounds in roots highlights an adaptive strategy in *A. venetum* to enhance antioxidant efficiency through regional specialization. By reallocating metabolic resources, the plant prioritizes energy and defense mechanisms in critical organs, achieving a balance between growth and stress response.

### 4.5. A Comparative Study of Growth Adaptation and Active Regulatory Mechanisms Under Saline and Alkaline Stress

Under different concentrations of NaCl and Na_2_CO_3_/NaHCO_3_ stress, the growth adaptation and active regulatory mechanisms of *A. venetum* exhibited similarities. Although salt stress and alkaline stress differ in certain aspects, with Na_2_CO_3_/NaHCO_3_ exerting a more significant inhibitory effect on the plant, the response strategies showed similar patterns between the two stresses. First, the plant regulates root structure and biomass allocation, investing more resources into root growth to enhance its adaptability to saline and alkaline environments. Secondly, changes in leaf anatomical structure, such as the thickening of the palisade tissue and alterations in surface structure, help reduce water loss and improve photosynthetic efficiency. In addition, under saline and alkaline stress, the plant synthesizes soluble sugars, proteins, and flavonoids to perform osmoregulation and antioxidant defense. Under both NaCl and Na_2_CO_3_/NaHCO_3_ stress, secondary metabolites in the plant exhibited similar accumulation patterns, particularly in the roots, where the accumulation of flavonoids significantly increased, indicating the crucial role of the roots in antioxidant defense against saline and alkaline stress. However, it is worth noting that alkaline stress has a more significant impact on sugars and proteins, leading to reduced sugar accumulation in the roots, which may also affect the integrity of the root membrane. This suggests that the stress response under alkaline conditions may differ from that under saline stress, and further research is needed to explore this aspect.

## 5. Conclusions

**Figure 9:**
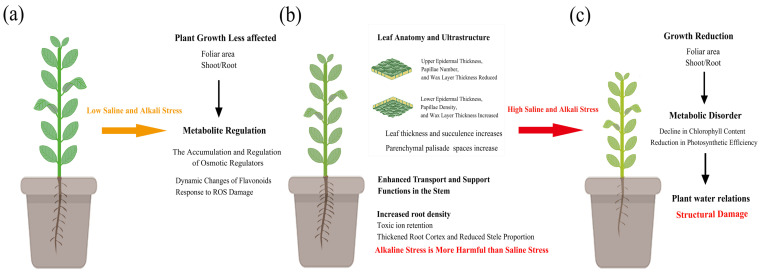
Effects of mild and severe saline stress and alkali stress on *A. venetum*. (**a**) Normal Growth; (**b**) Mild Stress; (**c**) Severe Stress. Under mild saline and alkali stress conditions, *A. venetum* exhibits strong compensatory ability. However, as the intensity of stress increases, the structural integrity of the plant is significantly compromised. Compared to saline stress, alkali stress has a more pronounced negative effect on the growth of *A. venetum*. Saline and alkali stresses primarily affect the above-ground parts of the plant, especially the leaves, where water balance is disrupted, leading to a decrease in relative leaf water content. As the stress intensity increases, chlorophyll content and chlorophyll fluorescence parameters show a parallel declining trend. At the same time, the accumulation of salt in the root zone reduces the osmotic potential, decreasing the availability of water. Despite these challenges, *A. venetum* mitigates the adverse effects of saline and alkali stress through adaptive mechanisms such as structural optimization and metabolic regulation.

This study demonstrates that *Apocynum venetum* L. exhibits considerable saline-alkali tolerance through coordinated regulation involving anatomical modifications and metabolite accumulation under saline and alkali stress. Specifically, the root system alleviates stress-induced damage and ensures water absorption and transport by means of cortical thickening, accumulation of osmotic regulatory substances, and increased xylem vessel diameter. The stem enhances water transport efficiency and provides structural support through modifications in xylem and phloem structures. Meanwhile, the leaf adapts to the stressful environment by thickening the palisade tissue and altering surface wax composition, thereby balancing photosynthetic capacity with water retention. Notably, the accumulation of soluble sugars, proteins, and flavonoids—particularly in the roots, which serve as the primary organ for stress mitigation—plays a critical role in osmotic adjustment and antioxidant defense (Figure 9). These findings reveal the adaptive strategies of *A. venetum* under salt-alkali stress and provide valuable insights for the development of *A. venetum* resources in saline-alkali soils, as well as a theoretical basis for the breeding of salt-tolerant crops.

## Data Availability

The datasets presented in this study are included in the main text; further inquiries can be directed to the corresponding author.

## Appendix A

**Table A1:** Anatomical parameters of *A. venetum* (*Apocynum venetum* L.) roots under saline stress and alkali stress. Data are presented as the mean ± SE from three replicates. Significant differences among treatments were determined using ANOVA and multiple range tests. Non-overlapping letters (a–f) indicate significance at the 95% confidence level.

Anatomical Parameters of Roots (µm)	CK (Control Group)	S1 (Nacl 80 mM)	S2 (Nacl 160 mM)	S3 (Nacl 240 mM)	A1 (Na_2_CO_3_/NaHCO_3_ 50/100 mM)	A2 (Na_2_CO_3_/NaHCO_3_ 100/50 mM)
Root Diameter	1387.17 ± 2.49 ^b^	1474.64 ± 3.65 ^a^	1346.79 ± 2.61 ^c^	1310.65 ± 3.90 ^d^	1158.69 ± 1.93 ^e^	1135.98 ± 3.83 ^f^
Epidermis Thickness	73.66 ± 2.55 ^a^	78.97 ± 0.86 ^a^	74.82 ± 1.75 ^a^	64.51 ± 2.73 ^b^	60.85 ± 1.20 ^b^	66.32 ± 0.95 ^b^
Cortex Thickness	248.11 ± 2.24 ^d^	287.57 ± 1.01 ^c^	306.51 ± 1.32 ^b^	312.10 ± 1.50 ^a^	249.18 ± 2.30 ^d^	285.11 ± 1.23 ^c^
Stele Diameter	727.63 ± 2.70 ^b^	776.30 ± 2.79 ^a^	626.99 ± 1.69 ^c^	612.13 ± 1.75 ^d^	608.64 ± 1.75 ^d^	459.04 ± 1.44 ^e^
Phloem Thickness	73.10 ± 1.14 ^a^	62.48 ± 1.29 ^b^	53.60 ± 0.80 ^c^	47.99 ± 0.83 ^d^	40.50 ± 1.29 ^e^	40.50 ± 0.58 ^e^
Xylem Thickness	643.99 ± 1.20 ^b^	717.50 ± 3.38 ^a^	548.94 ± 1.17 ^d^	526.10 ± 1.87 ^e^	556.39 ± 1.69 ^c^	364.84 ± 1.35 ^f^
Maximum Vessel Diameter	36.81 ± 0.92 ^e^	58.33 ± 0.40 ^c^	66.17 ± 0.92 ^b^	74.18 ± 1.62 ^a^	49.19 ± 0.31 ^d^	50.14 ± 0.61 ^d^

**Table A2:** Anatomical parameters of *A. venetum* stems subjected to saline stress and alkali stress. Values denote the mean ± SE from three replicates. Based on ANOVA and multiple range tests, distinct letters (a–f) signify significant discrepancies among treatments at a 95% confidence level.

Anatomical Parameters of Steams (µm)	CK (Control Group)	S1 (Nacl 80 mM)	S2 (Nacl 160 mM)	S3 (Nacl 240 mM)	A1 (Na_2_CO_3_/NaHCO_3_ 50/100 mM)	A2 (Na_2_CO_3_/NaHCO_3_ 100/50 mM)
Stem Diameter	1263.46 ± 3.53 ^f^	1284.24 ± 1.06 ^e^	1526.31 ± 1.58 ^c^	1873.25 ± 4.82 ^a^	1471.30 ± 4.01 ^d^	1579.02 ± 3.80 ^b^
Cortex Thickness	123.81 ± 2.79 ^e^	132.28 ± 1.17 ^d^	169.46 ± 2.67 ^c^	221.68 ± 2.76 ^a^	218.98 ± 3.15 ^a^	200.47 ± 2.12 ^b^
Phloem Thickness	58.33 ± 0.61 ^b^	58.54 ± 0.55 ^b^	35.06 ± 1.60 ^d^	42.80 ± 1.01 ^c^	57.01 ± 1.23 ^b^	62.87 ± 1.90 ^a^
Phloem Fiber Cell Wall Thickness	3.13 ± 0.09 ^d^	3.71 ± 0.06 ^c^	4.18 ± 0.06 ^b^	5.28 ± 0.12 ^a^	3.37 ± 0.06 ^d^	1.19 ± 0.07 ^e^
Endogenous Phloem Thickness	43.04 ± 1.69 ^d^	45.50 ± 1.14 ^d^	53.30 ± 1.14 ^c^	67.75 ± 2.73 ^a^	63.83 ± 1.11 ^b^	65.36 ± 2.13 ^ab^
Xylem Thickness	335.95 ± 2.61 ^f^	376.66 ± 5.59 ^e^	471.21 ± 2.09 ^c^	532.98 ± 2.61 ^a^	426.39 ± 2.33 ^d^	509.19 ± 2.09 ^b^
Maximum Vessel Diameter	43.41 ± 2.61 ^d^	52.07 ± 1.69 ^c^	64.19 ± 1.75 ^a^	66.93 ± 1.20 ^a^	56.37 ± 1.23 ^b^	57.53 ± 1.93 ^b^
Pith Diameter	229.36 ± 2.86 ^e^	285.14 ± 0.64 ^d^	346.54 ± 2.00 ^c^	451.72 ± 2.61 ^a^	362.81 ± 1.04 ^b^	282.34 ± 2.27 ^d^

**Table A3:** Anatomical parameters of *A. venetum* leaves under saline stress and alkali stress. Values represent the mean ± SE of three replicates. Based on ANOVA and multiple range tests, non-overlapping letters (a–f) indicate significant differences among treatments at a 95% confidence level.

Anatomical Parameters of Leafs (µm)	CK (Control Group)	S1 (Nacl 80 mM)	S2 (Nacl 160 mM)	S3 (Nacl 240 mM)	A1 (Na_2_CO_3_/NaHCO_3_ 50/100 mM)	A2 (Na_2_CO_3_/NaHCO_3_ 100/50 mM)
Average Leaf Thickness	156.50 ± 0.75 ^d^	157.52 ± 0.17 ^c^	164.94 ± 0.16 ^b^	176.85 ± 0.64 ^a^	158.78 ± 0.78 ^c^	139.42 ± 0.50 ^e^
Upper Epidermis Thickness	19.38 ± 0.30 ^a^	15.89 ± 0.20 ^b^	14.27 ± 0.11 ^c^	13.92 ± 0.21 ^c^	12.73 ± 0.27 ^d^	12.20 ± 0.33 ^d^
Lower Epidermis Thickness	14.47 ± 0.08 ^d^	16.41 ± 0.09 ^b^	18.10 ± 0.28 ^a^	15.44 ± 0.17 ^c^	16.19 ± 0.38 ^bc^	14.64 ± 0.27 ^d^
Wax Thickness of Upper Epidermis	1.62 ± 0.02 ^a^	1.41 ± 0.02 ^b^	1.40 ± 0.02 ^bc^	0.98 ± 0.02 ^d^	1.32 ± 0.03 ^c^	0.90 ± 0.01 ^e^
Wax Thickness of Lower Epidermis	1.41 ± 0.02 ^b^	1.49 ± 0.01 ^b^	1.86 ± 0.05 ^a^	1.25 ± 0.03 ^c^	1.47 ± 0.03 ^b^	1.13 ± 0.05 ^d^
Palisade Tissue Thickness	48.52 ± 0.47 ^f^	67.35 ± 0.38 ^c^	73.97 ± 0.23 ^b^	76.82 ± 0.23 ^a^	65.42 ± 0.35 ^d^	50.70 ± 0.54 ^e^
Spongy Tissue Thickness	67.80 ± 0.30 ^b^	50.02 ± 0.22 ^f^	63.53 ± 0.30 ^c^	71.02 ± 0.55 ^a^	61.82 ± 0.26 ^d^	57.99 ± 1.15 ^e^
Midvein Diameter	305.04 ± 0.30 ^c^	287.13 ± 0.79 ^d^	313.15 ± 0.55 ^b^	375.90 ± 0.90 ^a^	256.70 ± 0.67 ^e^	249.45 ± 0.44 ^f^
Xylem Thickness	34.80 ± 0.26 ^d^	36.17 ± 0.25 ^c^	38.83 ± 0.27 ^b^	43.17 ± 0.50 ^a^	29.33 ± 0.33 ^e^	29.90 ± 0.34 ^e^
Phloem Thickness	22.61 ± 0.54 ^a^	20.44 ± 0.44 ^b^	19.08 ± 0.20 ^c^	18.90 ± 0.38 ^c^	17.99 ± 0.02 ^d^	14.87 ± 0.12 ^e^
